# A strategy for emergency treatment of *Schistosoma japonicum*-infested water

**DOI:** 10.1186/1756-3305-4-209

**Published:** 2011-11-02

**Authors:** You-Sheng Liang, Wei Wang, Yun-Tian Xing, Hong-Jun Li, Yong-Liang Xu, Xue-Hui Shen, Guo-Li Qu, You-Zi Li, Jian-Rong Dai

**Affiliations:** 1 Jiangsu Institute of Parasitic Diseases, 117 Yangxiang, Meiyuan, Wuxi 214064, Jiangsu Province, People's Republic of China; 2 Key Laboratory on Technology for Parasitic Disease Prevention and Control, Ministry of Health, 117 Yangxiang, Meiyuan, Wuxi 214064, Jiangsu Province, People's Republic of China; 3 Dantu District Center for Disease Control and Prevention, 171 Guyang Avenue, Zhenjiang City, Danyang 212028, Jiangsu Province, People's Republic of China

**Keywords:** Schistosomiasis japonica, *Schistosoma japonicum*, cercaria, suspension concentrate of niclosamide, emergence treatment, infested water, the Yangtze River

## Abstract

**Background:**

Schistosomiasis japonica, caused by contact with *Schistosoma japonicum *cercaria-infested water when washing, bathing or production, remains a major public-health concern in China. The purpose of the present study was to investigate the effect of a suspension concentrate of niclosamide (SCN) on killing cercaria of *S. japonicum *that float on the water surface, and its toxicity to fish, so as to establish an emergency-treatment intervention for rapidly killing cercaria and eliminating water infectivity.

**Results:**

At 30 min after spraying 100 mg/L SCN, with niclosamide dosages of 0.01, 0.02, 0.03, 0.04 g/m^2^, the water infectivity reduced significantly and no infectivity was found at 60 min after spraying SCN. The surface of static water was sprayed with 100 mg/L SCN, the peak concentration was found at 0 min, and the solution diffused to site with a water depth of 10 cm after 10 min. 30 min later, SCN diffused to the whole water body, and distributed evenly. After spraying 100 mg/L SCN onto the surface of the water with a volume of(3.14 × 20^2^×50)cm^3^, with niclosamide dosages of 0.02 g/m^2^, 96 h later, no death of zebra fish was observed.

**Conclusions:**

By spraying 100 mg/L SCN, with a niclosamide dosage of 0.02 g/m^2 ^onto the surface of *S. japonicum*-infested water, infectivity of the water can be eliminated after 30-60 min, and there is no evident toxicity to fish. This cercaria-killing method, as an emergency-treatment intervention for infested water, can be applied in those forecasting and early warning systems for schistosomiasis.

## Background

Schistosomiasis japonica remains a major public-health concern in China [1-8]. Concerted control effects since the 1950s have dramatically reduced the number of areas endemic for the parasite as well as the burden of disease among humans, and the remaining core endemic regions are mainly located in the five provinces of Hunan, Hubei, Jiangxi, Anhui and Jiangsu along the middle and lower reaches of the Yangtze River [4-6,9-13]. Nevertheless, following the effect of flooding of the upper reaches of the Yangtze River during the annual monsoon season [14-18], the schistosomiasis epidemic is still severe in some endemic areas. Acute human schistosomiasis cases are detected each year and over 0.7 million people are estimated to be infected, with a further 30 million at risk of infection [1,12,19-21]. It is critical to establish an effective, sensitive monitoring network for infested water and then rapidly treat the infested water, so as to interrupt or control the spread and outbreak of the disease, restrict the epidemic, and reduce the damage of disease on human health. Therefore, surveillance, forecast and response, inseparable from monitoring and forecast systems of infectious diseases, are all indispensable. A field surveillance system and forecast platform of schistosomisis has been established in high risk regions along the Yangtze River in China, to monitor the water infectivity of the Yangtze River during the period of schistosomiasis transmission [22-24]. It is, therefore, of great importance to establish a simple and rapid emergency treatment approach to eliminate the water infectivity following the detection of *S. japonicu*m-infested water.

Schistosomiasis is caused by contact with cercaria-infested water when washing, bathing or production [25]. Unlike mature cercariae of *S. mansoni *and *S. haematobium *that suspend evenly in water after shedding from the snail intermediate hosts, more than 98% of the mature cercaria of *S. japonicum *float on the water surface [26,27], which provides the possibility and feasibility for killing *S. japonicum *cercaria on the surface of the water. Besides killing *Oncomelania. hupensis*, niclosamide also shows high activity against cercariae of *S. japonicum *[28,29]. However, it is difficult to dissolve in water and toxic to aquatic animals such as fish, which limits its use for cercaria-killing in the field. To try to overcome these problems, a 25% suspension concentration of niclosamide (SCN), a novel formulation of niclosamide, was developed [30]. The formulation has good dispersion and suspension and is suitable for spraying at a high concentration directly with a sprayer in various environments (such as marshland and mountainous areas). As SCN can be mixed with water in any ratio, it is more suitable for use than the preparation of 50% wettable powder of niclosamide ethanolamine salt (WPN), the current most widely used molluscicide in China [31]. The present study made use of the biological characteristic of *S. japonicum *cercaria floating on the water surface and the chemical characteristic of rapid dispersion of SCN in water, to kill cercariae rapidly but without toxicity to aquatic animals by using high concentrations of SCN sprayed onto the water surface.

## Materials and methods

### Study area

The field experiment was focused on Renmin river in Dantu District of Zhenjiang City, China, a schistosomiasis-endemic area, measuring 1100 × 5 m. An area of 5000 m^2 ^was detected for the habitat of *S. japonicum*-infected *O. hupensis *snails.

### Mice

Mice of the Kunming strain, each weighing 20~30 g, were purchased from Shanghai SLAC Laboratory Animal Co. LTD. (Shanghai, China), and were given free access to food and water.

### *S. japonicum *cercaria

The cercariae of *S. japonicum *were obtained from naturally infected *O. hupensis *collected from Anhui Province, China. They were exposed to artificial light for 3 h in de-chlorinated water at a temperature of 28°C. All cercariae were used within 30 min after shedding to maintain the activity.

### Zebra fish

Two-month old *Brachydanio rerio Hamiton *weighing 0.28 ± 0.15 g were purchased in a local market and maintained for 3 days in the laboratory prior to use.

### Suspension concentrate of niclosamide (SCN)

SCN (batch no. 200402) was manufactured by Jiangsu Institute of Materia Medica (Nanjing, China), and had a purity of 25%.

### Mouse cages

Cages for raising mice were set up in the field. Each cage was a cuboid measuring 65 × 7 × 7 cm, made of a strong frame of iron wire covered with wire netting (with a mesh size of less than 15 mm), and divided into 5 parts. Both ends of the cage were bound to foam packages (size of 35 × 10 × 10 cm), making the cages exhibiting a "I" -type.

### Determination of niclosamide sample using high performance liquid chromatography (HPLC)

1 mL of each water sample was analyzed by the HPLC system (Waters Corporation, USA) to determine the assay of niclosamide. The concentration was calculated by the following formula:

$$C=A_{1}\times C_{s}∕A_{2}$$

Where *A*_1 _and *A*_2 _are the average values of peak area of niclosamide in standard solution and water samples, respectively, *C*_s _is the concentration of niclosamide in standard solution.

### Screening of spraying concentrations of SCN

SCN was diluted with de-chlorinated water and formulated into solutions of 1000, 100, 10, 1 and 0.1 mg/L. De-chlorinated water (at a temperature of 28°C) was added into cylindrical buckets (at a diameter of 7 cm, surface area of about 38.47 cm^2^), each containing 30 ml and then 200 freshly released cercaria of *S. japonicum *were transferred to the buckets. The SCN solutions at 5 concentrations (1000, 100, 10, 1 and 0.1 mg/L), each containing 3.5 ml, were sprayed onto the water surface with a portable mini nebulizer (Golden Spring Plastic Co., Ltd, China). At 0, 10 and 30 min after spraying, a mouse was put into each bucket for 30 min, and then transferred to the laboratory and raised. A total of 10 mice were used for each concentration and each time point. De-chlorinated water served as an untreated control.

### Screening of spraying values of SCN

De-chlorinated water (at a temperature of 28°C) was dispensed into five rectangular glass jars (at a length of 38.5 cm and width of 24 cm), at water depths of 10, 20, 30, 40 cm (experimental groups) and 20 cm (control). Freshly shed cercariae of *S. japonicum *were then placed onto the water surface of the glass jars, each containing 2000 cercariae. SCN solutions at a concentration of 100 mg/L were sprayed based on the niclosamide dosages of 0.01, 0.02, 0.03 and 0.04 g/m^2^, for killing the cercaria, using a portable mini nebulizer, the control was not treated with SCN. At 30 and 60 min after spraying, iron cages, each containing 10 mice, were suspended on the water surface, 30 min later, mice were transferred to the laboratory and raised.

### Field experiment of spraying SCN to kill cercaria

Sixty mice were placed into 6 mouse cages, each containing 10 mice, and divided into 2 groups of prior-spraying SCN and post-spraying SCN. In September, all mice in the prior-spraying group were set up on the water surface of the study area for 4 h per day (9:00-13:00) with a successive 2 days, and the interval between cages was 20 m. Two days later, 100 mg/L SCN solutions were sprayed on the water surface at a niclosamide dosage of 0.02 g/m^2 ^with a large sprayer, 1 h later, mice in the post-spraying group were set up on the water surface for 4 h per day (9:00-13:00) with a successive 2 days, and the aquatic animals such as fish that died were counted and recorded. Following the field experiment, all mice were then put into cages and transferred to the laboratory and raised.

For all of the above 3 experiments, the mice were killed 35 days later, and adult *S. japonicum *worms in the hepatic and portomesenteric veins were recovered by perfusion and counted. The reductions in the total number of worms recovered that were apparently caused by drug treatment were then estimated, as percentages, by comparison with the numbers of worms recovered from the untreated control group. The statistical analysis was performed in SPSS 13.0 (SPSS Inc., Chicago, IL, USA). Statistical significance of each reduction was then estimated using Fisher's least significant-difference (LSD) tests, with a P-value < 0.05 considered indicative of a statistically significant difference.

### SCN dispersion and toxicity to fish

A cylindrical bucket (at a diameter of 40 cm and height of 50 cm) was filled with de-chlorinated water (at a temperature of 28°C), and then 50 zebra fish were transferred into the bucket. SCN with a concentration of 100 mg/L was sprayed evenly on the surface of the static water at niclosamide dosages of 0.01, 0.02, 0.03 and 0.04 g/m^2^. At 0, 10, 30 and 60 min after spraying, a 1 ml water sample was collected at water depths of 0, 10, 20, 30 and 40 cm, respectively. Niclosamide in the water sample was determined using HPLC, and the zebra fish were observed at 0, 24, 48, 72 and 96 h. Any fish that died were immediately removed and counted.

## Results

### Effect of SCN spraying with different drug concentrations on water infectivity

SCN solutions at concentrations of 1000, 100, 10, 1 and 0.1 mg/L, each being 3.5 ml, were sprayed on the water surface, respectively, and then mice were transferred immediately for determination of the water infectivity. It was found that the total worm burdens of mice infected from water treated with SCN were reduced significantly in comparison to the untreated controls (all *P *values < 0.01 The total worm burdens reduced as the drug concentrations increased. The total worm burden of mice from the water treated with 1000 mg/L SCN 10 min after spraying reduced to 0, and compared with the untreated control, the total worm burdens of mice infected from water treated with SCN were all significantly reduced (all *P *values < 0.01). 30 min after spraying, both of the total worm burdens of mice following treatment of water with 1000 and 100 mg/L SCN reduced to 0. Significant reductions in total worm burdens were found in mice infected from water treated with SCN compared with the control (all *P *values < 0.01) (Table 1). It is indicated that following spraying with 100 mg/L SCN onto the water surface for 30 min, the infectivity is eliminated.

**Table 1:** Worm burdens of infected mice after the spraying of different concentrations of SCN on water surface

Drug concentration (mg/L)	Infected immediately after spraying		Infected at 10 min after spraying		Infected at 30 min after spraying	
	
	No. mice dissected	Worm burdens (Mean ± SD)	No. mice dissected	Worm burden(Mean ± SD)	No. mice dissected	Worm burdens (Mean ± SD)
1000	10	15.5 ± 11.7	10	0	10	0
100	10	56.0 ± 26.8	10	3.2 ± 3.0	10	0
10	10	78.5 ± 28.4	10	10.7 ± 11.9	10	22.0 ± 16.3
1	10	73.4 ± 24.6	10	56.6 ± 21.8	9	69. 6 ± 22.7
0.1	10	70.7 ± 13.7	10	60.9 ± 26.4	10	80.0 ± 22.7
0 (Control)	10	103.0 ± 14.1	10	111.2 ± 19.0	9	119.4 ± 11.3

### Effect of SCN with different niclosamide dosages on water infectivity

SCN solutions with a concentration of 100 mg/L were sprayed on the surface of the water (water depths of 10, 20, 30 and 40 cm) at niclosamide dosages of 0.01, 0.02, 0.03 and 0.04 g/m^2^, 30 min after spraying, mice were placed on the water surface for determination of infection, the total worm burdens of mice infected from water treated with four dosages of SCN reduced significantly compared to the untreated control (all *P *values < 0.01), and the water infectivity was markedly less. 60 min later, all total worm burdens of mice infected from water treated with SCN reduced to 0, and the water infectivity was completely eliminated (Table 2).

**Table 2:** Worm burdens of infected mice at different water depths and different times after spraying 100 mg/L SCN with different niclosamide dosages on water surface

Water depth (cm)	SCN with different niclosamide dosage (g/m^2^)	Infected at 30 min after spraying		Infected at 60 min after spraying	
		
		No. mice dissected	Worm burdens (Mean ± SD)	No. mice dissected	Worm burdens (Mean ± SD)
10	0.01	10	3.2 ± 3.9	10	0
20	0.02	9	0.1111 ± 0.3333	10	0
30	0.03	9	0.7778 ± 1.3017	9	0
40	0.04	8	1.1251 ± 1.2464	8	0
20	0 (control)	10	30.457 ± 11.428	10	29.5714 ± 12.7129

### Effect of spraying SCN on *S. japonicum *cercaria in field

The infection rate of mice was 33.33% (9/27) prior to spraying SCN, with mean worm burdens of 1.63 worms per mouse. Following spraying 100 mg/L SCN on the water surface at a niclosamide dosage of 0.02 g/m^2^, no infected mice were detected (0/30). There were also no dead aquatic animals, such as fish, observed after spraying during a period of 2 days.

### SCN dispersion and toxicity to fish

SCN solutions with a concentration of 100 mg/L were sprayed on the surface of the static water at niclosamide dosages of 0.01, 0.02, 0.03 and 0.04 g/m^2^, and the concentration of SCN on the water surface reached the maximum at 0 min, being 0.07, 0.24, 0.31 and 0.36 mg/L, respectively. 10 min later, SCN dispersed to the site with a water depth of 30 cm and 30 min, it would spread to the whole waterbody, and niclosamide would be dispersed evenly in the water body (Table 3).

**Table 3:** Concentrations of SCN at different water depths after spraying 100 mg/L SCN with different niclosamide dosages on water surface (mg/L)

Spraying SCN with different niclosamide dosage on water surface (g/m^2^)	Time (min)	At: water depth (cm)				
		
		0(water surface)	10	20	30	40
0.01	0	0.070	0.000	0.000	0.000	0.000
	10	0.000	0.000	0.000	0.000	0.000
	30	0.000	0.000	0.000	0.000	0.000
	60	0.000	0.000	0.000	0.000	0.000
0.02	0	0.240	0.000	0.000	0.000	0.000
	10	0.048	0.042	0.039	0.039	0.000
	30	0.046	0.045	0.044	0.045	0.045
	60	0.045	0.046	0.044	0.045	0.047
0.03	0	0.314	0.000	0.000	0.000	0.000
	10	0.070	0.063	0.058	0.059	0.000
	30	0.080	0.077	0.081	0.080	0.084
	60	0.078	0.080	0.078	0.078	0.083
0.04	0	0.360	0.000	0.000	0.000	0.000
	10	0.134	0.085	0.078	0.080	0.000
	30	0.108	0.103	0.109	0.108	0.104
	60	0.097	0.106	0.098	0.103	0.100

SCN solutions with a concentration of 100 mg/L were sprayed on the water (measuring 3.14 × 20^2^×50 cm^3^) surface at niclosamide dosages of 0.01 and 0.02 g/m^2^, the mortality of zebra fish was 0 after spraying during a period of 96 h. When the dosage reached 0.03 g/m^2^, dead zebra fish were observed after 48 h (Table 4).

**Table 4:** Mortality rate of zebra fish after spraying 100 mg/L SCN with different dosages of niclosamide on water surface

Time (h)	SCN with different dosages of niclosamide (%, *n*/*N*)			
	
	0.01 g/m^2^	0.02 g/m^2^	0.03 g/m^2^	0.04 g/m^2^
0	0(0/50)	0(0/50)	0(0/50)	2 (1/50)
24	0(0/50)	0(0/50)	0(0/50)	10 (5/50)
48	0(0/50)	0(0/50)	4 (2/50)	16 (8/50)
72	0(0/50)	0(0/50)	8 (4/50)	20 (10/50)
96	0(0/50)	0(0/50)	14 (7/50)	24 (12/50)

## Discussion

Our findings showed, after screening and optimization of the spraying concentrations and dosages, the goal of rapid, effective cercariae-killing and low-toxicity to fish by spraying SCN onto the water surface for emergency treatment of *S. japonicum*-infested water can be achieved. It has been reported that the effective concentration of niclosamide to kill *S. japonicum *cercariae was 0.06-0.12 mg/L [29-33], and the non lethal concentration (LC_0_) of SCN to fish was 0.227 mg/L [34]. The present study indicated, SCN was formulated into a solution of 100 mg/L, and then sprayed on the surface of the *S. japonicum*-infested water with a niclosamide dosage of 0.02 g/m^2^, the effective concentration of niclosamide on the water surface reached 0.24 mg/L at 0 min, which was twice as much as the effective concentration for killing cercariae. It was also confirmed by experimental mouse infection, that the cercariae were killed and the water infectivity eliminated some 30-60 min after spraying SCN. HPLC showed that 10 min after spraying SCN had dispersed to the site with a water depth of 30 cm, and the drug concentrations quickly reduced to 0.039-0.048 mg/L for the water layer of 0-30 cm, which were much less than the LC_0 _of SCN to fish. No dead fish were found up to 96 h after spraying, which suggests that the approach had no toxic effects on fish. Both of the experiments in the laboratory and the field proved that, if 100 mg/L SCN is sprayed on the surface of the infested-water with a niclosamide dosage of 0.02 g/m^2^, 30-60 min later, the water infectivity will be eliminated and it is not toxic to fish. On the one hand, the drug with high concentrations contacts with the floating cercaria directly, and thus kills the cercaria rapidly, on the other hand, due to good dispersion, SCN is diluted and spreads quickly in water, and the concentration rapidly decreases, the resultant lessened toxicity to aquatic organisms alleviates or avoids damage to the aquatic animals, but remains effective against cercaria.

Currently in China, schistosomiasis japonica mainly remains endemic in lake regions of five provinces of Hunan, Hubei, Jiangxi, Anhui and Jiangsu along the lower and middle reaches of the Yangtze River [7,12,35-37]. The water of the Yangtze River rises every spring and summer flood period. Following the marshland along the Yangtze River flooded by the rise in water level, the *S. japonicum*-infected snails on the marshland are very likely to release cercariae. At the same time, humans and domestic animals frequently come into contact with cercaria-infested water due to the needs of life and cultivation as well as the effect of flood disaster, resulting in increased probability of infections with *S. japonicum *[14,16-18]. It is, therefore, the high-occurrence season for *S. japonicum *transmission in China from May to September [16]. In order to monitor the changes in the endemic situation timely and effectively, based on the recent endemic data of schistosomiasis, a total of 45 forecast and surveillance sites, were screened and established, in those water bodies with snail distribution and frequent activities of humans and domestic animals, along the 200-km bank of the Yangtze River [22]. From May to September, a systematic and successive longitudinal surveillance was carried out in the forecast and surveillance sites by using sentinel mice. The infective water was published for forecast and prediction of high-risk settings, and then the emergency response system was operated, an integrated prevention and control approach was implemented for rapid elimination of the water infectivity, to remove the hidden dangers and reduce the damage of the epidemic spread to the minimum [23,24]. It is generally considered that, the elimination of water infectivity should include checking and control of infected snails, as well as cercaria-killing. However, due to the high water level in the flood period and consequent flooded marshlands, it is very difficult to check and kill the infected snails under water [16]. Thus to eliminate the water infectivity, killing the cercaria floating on the water surface is the most direct way.

## Conclusions

The established cercaria-killing strategy described here using high concentrations of SCN, has advantages of rapid treatment, easy operation, low toxicity to fish and good anti-cercaria effects, it is suitable for the emergency treatment of infested water in the flood period of the Yangtze River. In addition, it would play an important role particularly as an emergency treatment intervention for infested water in the prevention of acute schistosomiasis, cercaria control in large fish ponds, and emergency treatment of infested water in anti-flood strategies. More studies should be carried out to further verify the value of spraying with SCN.

## Competing interests

The authors declare that they have no competing interests.

## Authors' contributions

YSL and JRD conceived and designed the study and collected the data. YSL, WW, YTX, HJL, YLX, XHS, GLQ, YZL and JRD implemented the study. WW, YTX and YSL carried out the statistical analysis and prepared the manuscript. YSL, WW and JRD revised and finalized the manuscript. All of the authors read and approved the final version of the manuscript.
